# Supplementary material for: Ki-67 promotes inflammatory signaling governing neutrophil recruitment during respiratory infections
Source: EMBO Mol Med. 2025 Jun 10;17(8):2011–39. doi: 10.1038/s44321-025-00261-z (PMC12340004; doi:10.1038/s44321-025-00261-z)
Supplement: Supplementary file 12 — Expanded View Figures [file 44321_2025_261_MOESM12_ESM.pdf]

Expanded View Figures

Figure EV1. Identification of alveolar cells expressing Ki-67.

(**A**) Lungs of adult mice exposed to room air or hyperoxia as neonates were disassociated and stained for Ki-67 and VE-cadherin (CD144), PECAM (CD31) or CD45. (**A**) FACS plots show hyperoxia does not increase Ki-67 in CD144$^+$ or CD31$^+$ endothelial cells. $n = 5$ mice per plot. (**B**) FACS plots showing Ki-67 was not detected in CD45+ leukocytes. $n = 5$ mice per plot. (**C**) Lungs of adult *Sftpc*^{EGFP} mice exposed to room air or hyperoxia as neonates were immunostained for Ki-67 (red), EGFP (green), and counterstained with DAPI (blue). Arrow points to rare EGFP$^+$ AT2 cell expressing Ki-67. The proportion of EGFP$^+$ AT2 cells that also express Ki-67 were quantified and graphs as mean ± standard deviation. $n = 10$ mice per group. (Room air vs Hyperoxia: Not significant (NS)). (**D**) Lungs of adult Sftpc^{EGFP} mice exposed to room air or hyperoxia were stained for phospho-histone H3 (Ser10) (red), EGFP (green), and counterstained with DAPI (blue). Arrow points to a phospho-histone H3 (Ser10)$^+$ cell that was rarely detected in mice exposed to room air or hyperoxia. Note that the phospho-histone H3 (Ser10) staining could not be done on the same tissues stained for Ki-67 because both antibodies were made in the same species. The proportion of pHH3+ to EGFP+ cells were quantified and graphed as mean ± standard deviation. $n = 4$ mice per group. (Room air vs Hyperoxia: NS= not significant). Scale bar in (**C**, **D**) = 50 µm. Data reflects biological replicates analyzed by Student's t test (**C**, **D**).

© The Author(s)

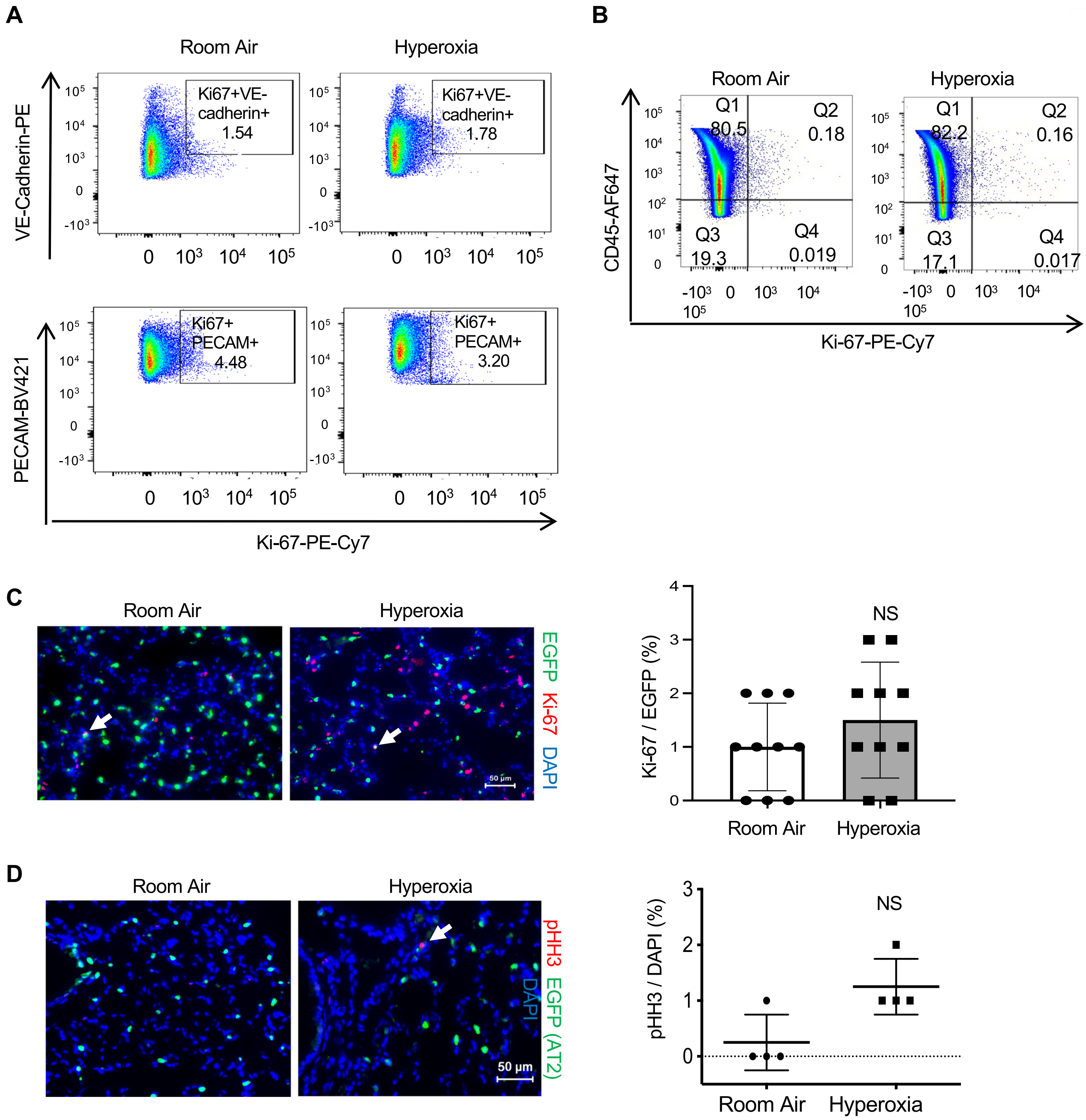
A
Room Air
Hyperoxia
VE-Cadherin-PE
Ki67+VE-cadherin+ 1.54
Ki67+VE-cadherin+ 1.78
PECAM-BV421
Ki67+ PECAM+ 4.48
Ki67+ PECAM+ 3.20
Ki-67-PE-Cy7
B
Room Air
Hyperoxia
CD45-AF647
Q1 80.5
Q2 0.18
Q3 19.3
Q4 0.019
Q1 82.2
Q2 0.16
Q3 17.1
Q4 0.017
Ki-67-PE-Cy7
C
Room Air
Hyperoxia
EGFP Ki-67 DAPI
50 µm
Ki-67 / EGFP (%)
NS
Room Air
Hyperoxia
D
Room Air
Hyperoxia
pHH3 EGFP (AT2) DAPI
50 µm
pHH3 / DAPI (%)
NS
Room Air
Hyperoxia

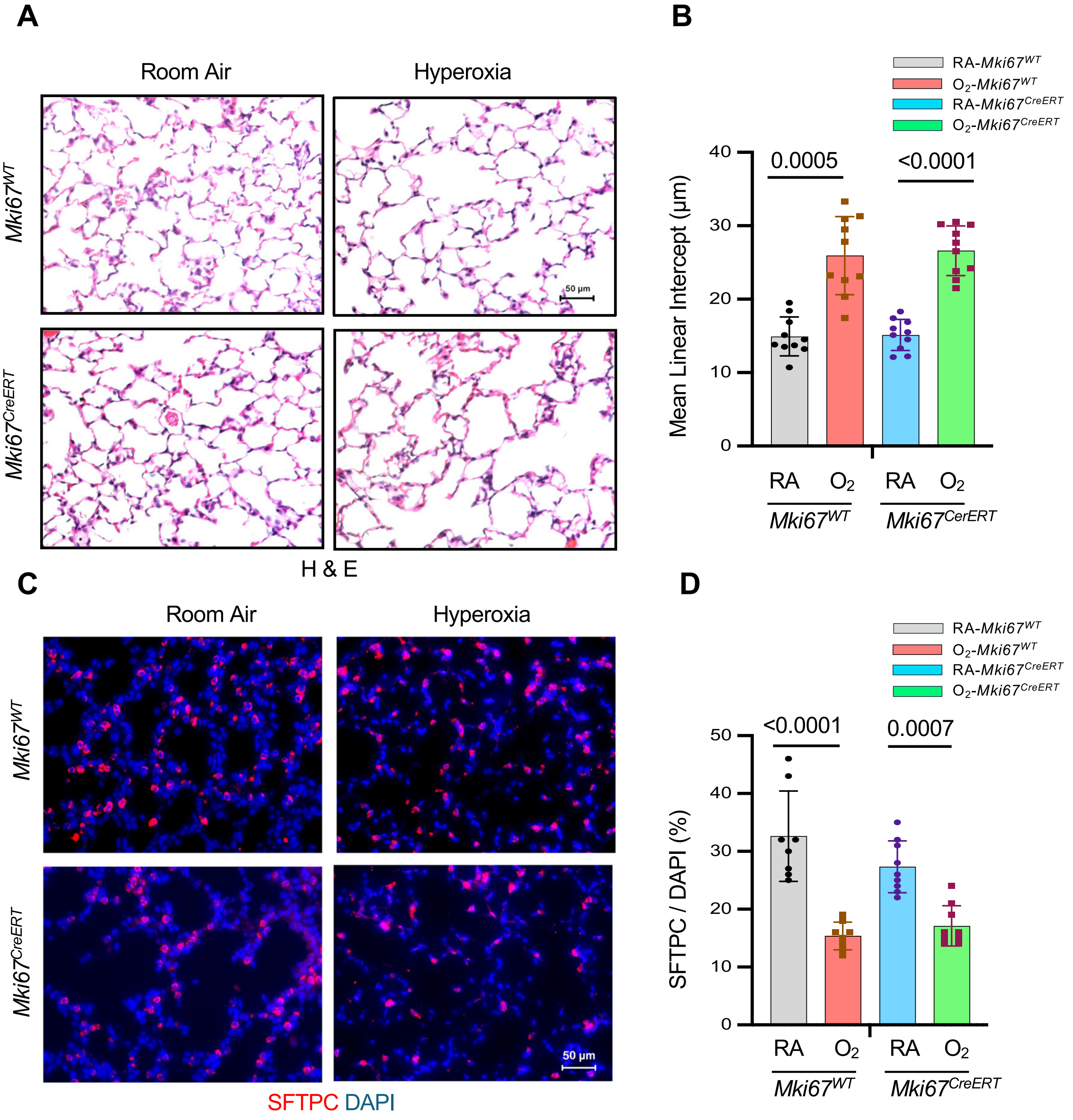

Figure EV2. Ki-67 is not required for neonatal hyperoxia to disrupt alveolar development.

(**A**) H&E stains of lungs from adult *Mki67*^{WT} and *Mki67*^{CreERT} hypomorph mice exposed to room air or hyperoxia. (**B**) Mean linear intercept of alveolar size in µm was measured in lungs of adult *Mki67*^{WT} and *Mki67*^{CreERT} hypomorph mice exposed to room air or hyperoxia and graphed as mean ± standard deviation. $n = 10$ mice per group. (RA-*Mki76*^{WT} vs O_2 *Mki67*^{WT}: $P = 0.0005$; RA *Mki67*^{CreERT} vs O_2-*Mki67*^{CreERT}; $P < 0.0001$). (**C**) Lungs from adult *Mki67*^{WT} and *Mki67*^{CreERT} hypomorph mice exposed to room air or hyperoxia were stained for SFTPC (red) and counterstained with DAPI (blue). (**D**) The proportion of SP-C^{+} to DAPI^{+} alveolar cells were quantified and graphed as mean ± standard deviation. $n = 9$ mice per group or 8 for *Mki67*^{WT} mice exposed to room air.(RA *Mki67*^{WT} vs O_2 *Mki67*^{WT}: $P < 0.0001$; RA *Mki67*^{CreERT} vs O_2-*Mki67*^{CreERT}; $P = 0.0007$). Data in (**B**, **D**) are graphed as mean ± SD with individual samples shown as circles or squares. Scale bar in (**A**, **C**) = 50 µm. Data reflect biological replicates analyzed by one-way ANOVA using Tukey-Kramer HSD (**B**, **D**).

© The Author(s)

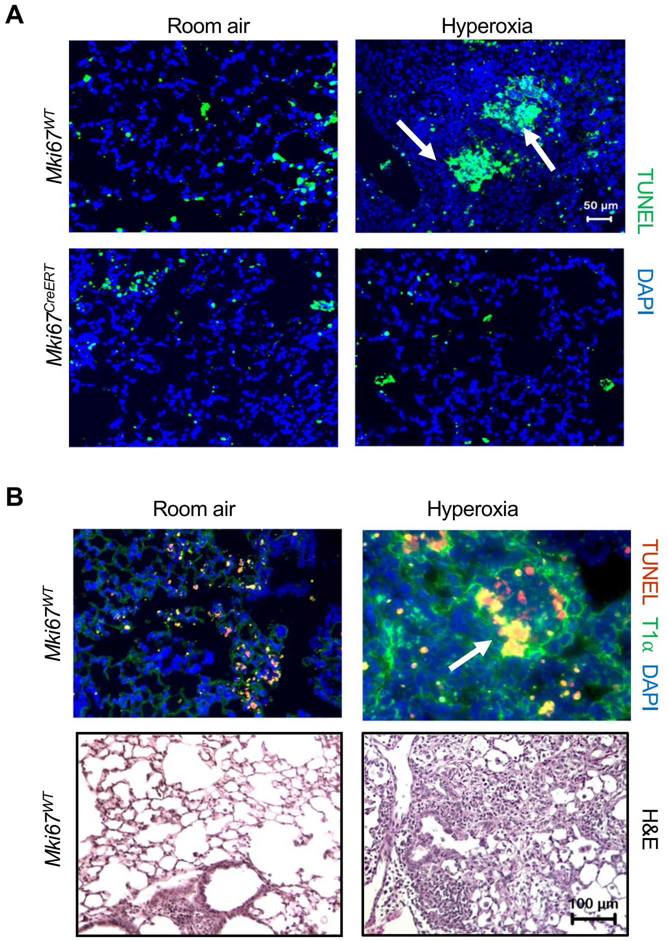

Figure EV3. Neonatal hyperoxia enhances AT1 apoptosis and NETosis in lungs of infected *Mki67^{WT}* but not *Mki67^{CreERT}* hypomorphs.

Adult *Mki67^{WT}* and *Mki67^{CreERT}* mice exposed to room air or hyperoxia were infected with Hkx31 IAV. (**A**) Lung sections collected on post-infection day 5 were stained with a TUNEL assay (green) used to detect DNA strand breaks and counterstained with DAPI (blue). (**B**) Adult *Mki67^{WT}* exposed to room air or hyperoxia were infected with Hkx31 IAV. Lung sections collected on post-infection day 5 were stained for TUNEL (red), antibody to T1α (green) used to detect AT1 cells, and counterstained with DAPI. Arrows point to patches of TUNEL+ cells with pseudo-yellow color in (**B**) reflecting TUNEL and T1α double-positive cells indicative of AT1 cell death. Images are representative of 10 mice per group with similar pathology. Scale bar in (**A**) = 100 µm, and (**B**) = 50 µm.

 © The Author(s)

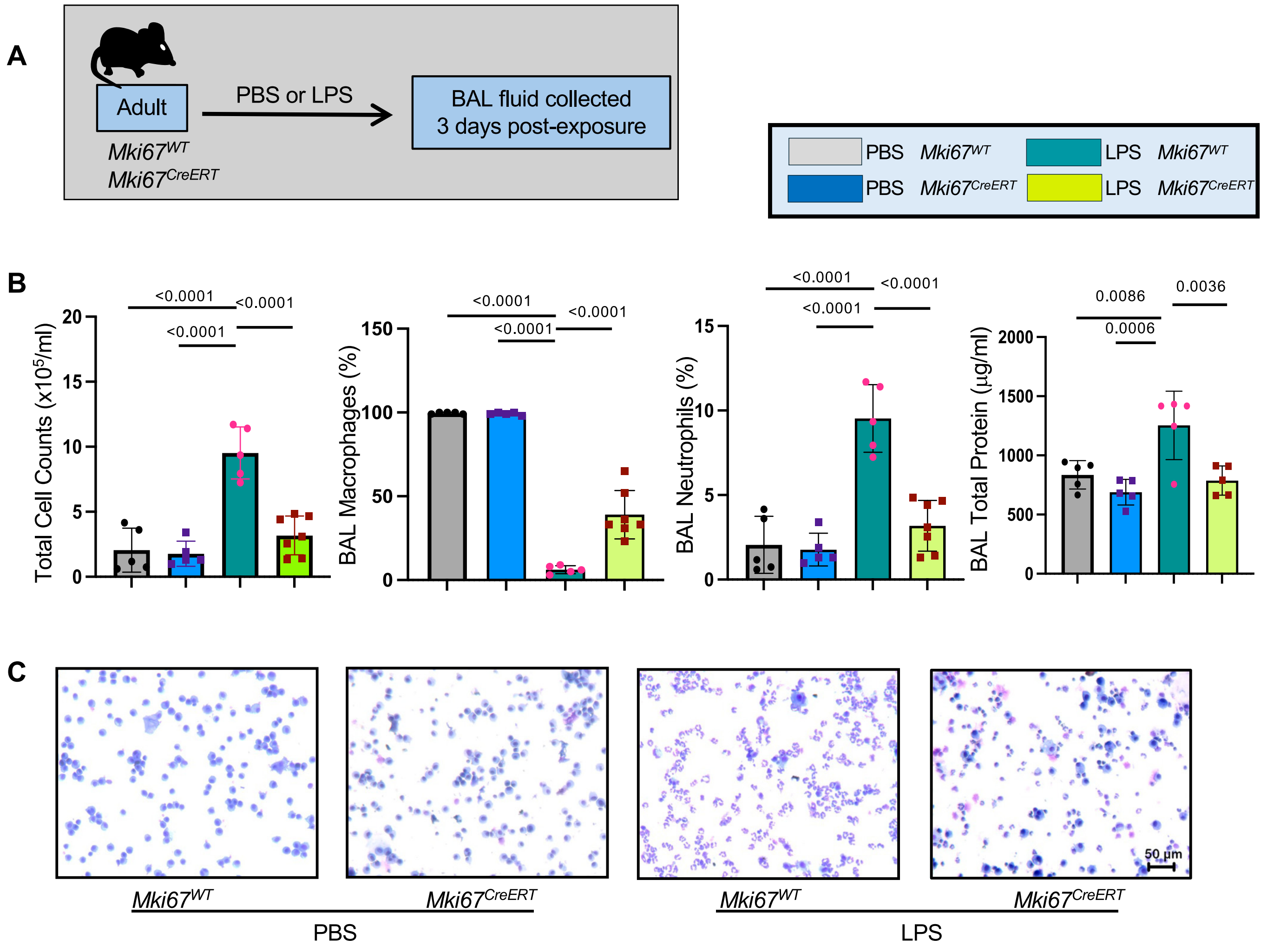

Figure EV4. Lung neutrophil recruitment and injury is attenuated in $Mki67^{CreERT}$ hypomorphs exposed to LPS.

(**A**) Bronchoalveolar lavages were performed 3 days after LPS (5 mg/kg) or PBS was instilled intratracheally into lungs of adult $Mki67^{WT}$ and $Mki67^{CreERT}$ hypomorph mice. (**B**) The total number of leukocytes, the proportion of macrophages, the proportion of neutrophils, and total protein were quantified in BAL fluid, and graphed as mean ± standard deviation with individual mice shown in circles or squares. $n = 5$ mice for all groups except 7 $Mki67^{CreERT}$ exposed to LPS. (Total Cell Counts: PBS-$Mki67^{WT}$ vs LPS-$Mki67^{WT}$: $P < 0.0001$; PBS-$Mki67^{CreERT}$ vs LPS-$Mki67^{WT}$: $P < 0.0001$; LPS-$Mki67^{WT}$ vs LPS-$Mki67^{CreERT}$: $P < 0.0001$). (Percent macrophages: PBS-$Mki67^{WT}$ vs LPS-$Mki67^{WT}$: $P < 0.0001$; PBS-$Mki67^{CreERT}$ vs LPS-$Mki67^{WT}$: $P < 0.0001$; LPS-$Mki67^{WT}$ vs LPS-$Mki67^{CreERT}$: $P < 0.0001$). (Percent neutrophils: PBS-$Mki67^{WT}$ vs LPS-$Mki67^{WT}$: $P < 0.0001$; PBS-$Mki67^{CreERT}$ vs LPS-$Mki67^{WT}$: $P < 0.0001$; LPS-$Mki67^{WT}$ vs LPS-$Mki67^{CreERT}$: $P < 0.0001$). (Total Protein: PBS-$Mki67^{WT}$ vs LPS-$Mki67^{WT}$: $P = 0.0086$; PBS-$Mki67^{CreERT}$ vs LPS-$Mki67^{WT}$: $P = 0.0006$; LPS-$Mki67^{WT}$ vs LPS-$Mki67^{CreERT}$: $P = 0.0036$). (**C**) Representative images of BAL cytospins obtained from the mice. Data in (**B**) graphed as mean ± standard deviation. Scale bar in (**C**) = 50 µm. Data reflects biological replicates analyzed by one-way ANOVA using Tukey-Kramer HSD (**B**).

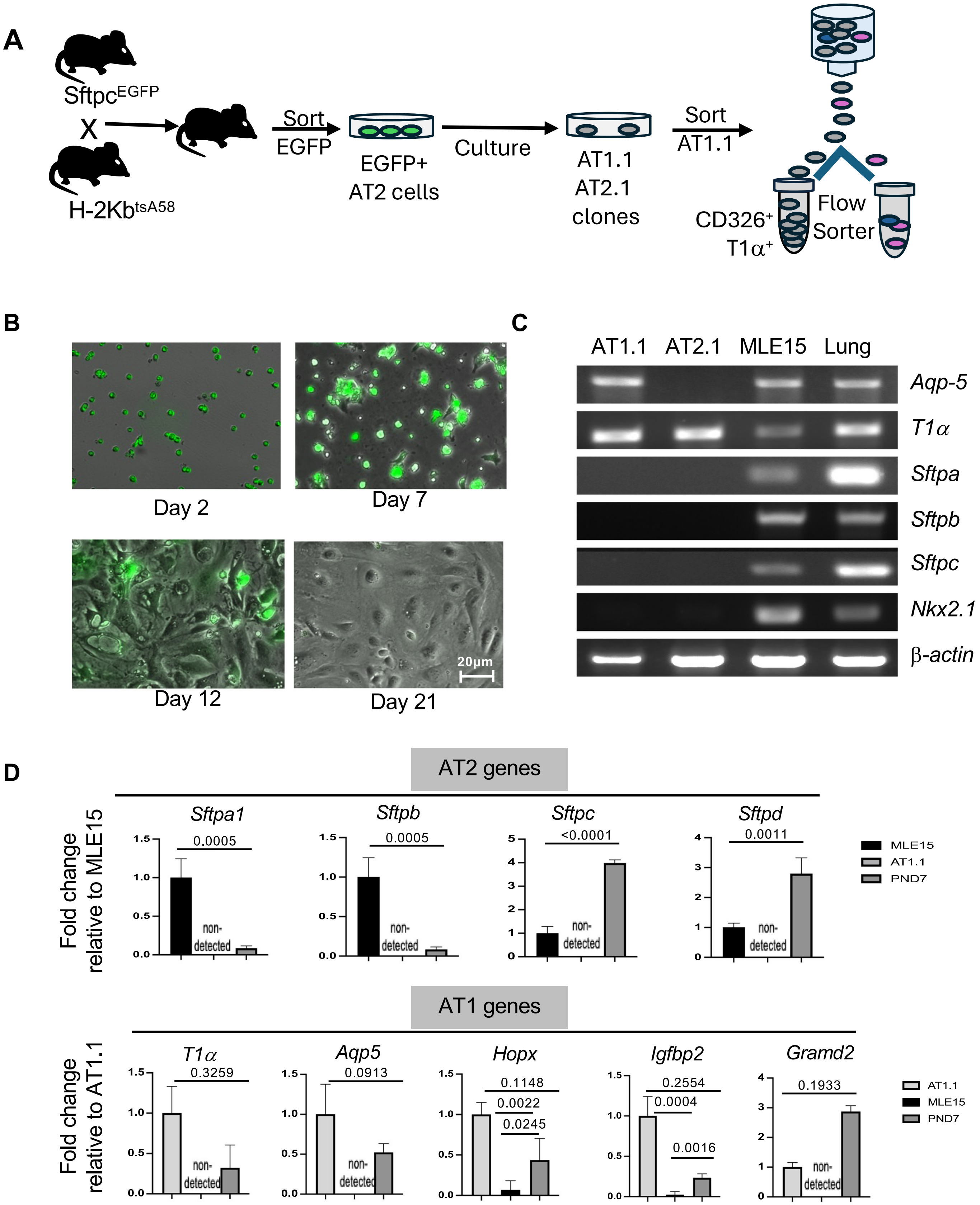
A
SftpcEGFP
X
H-2KbtsA58
Sort
EGFP
EGFP+
AT2 cells
Culture
AT1.1
AT2.1
clones
Sort
AT1.1
CD326+
T1α+
Flow
Sorter
B
Day 2
Day 7
Day 12
Day 21
20μm
C
AT1.1 AT2.1 MLE15 Lung
Aqp-5
T1α
Sftpa
Sftpb
Sftpc
Nkx2.1
β-actin
D
AT2 genes
Fold change relative to MLE15
Sftpa1
0.0005
non-detected
Sftpb
0.0005
non-detected
Sftpc
<0.0001
non-detected
Sftpd
0.0011
non-detected
MLE15
AT1.1
PND7
AT1 genes
Fold change relative to AT1.1
T1α
0.3259
non-detected
Aqp5
0.0913
non-detected
Hopx
0.1148
0.0022
0.0245
Igfbp2
0.2554
0.0004
0.0016
Gramd2
0.1933
non-detected
AT1.1
MLE15
PND7

 © The Author(s)

◀ **Figure EV5. Creation and characterization of mouse AT1.1 cell line.**

(**A**) Cartoon model showing how mouse AT1.1 cells were created from EGFP + AT2 cells isolated from Immortomice. (**B**) Images showing loss of green fluorescence as EGFP+ AT2 cells from Immortomice were cultured. (**C**) PCR was used to detect *Aqp5*, *T1a*, *Sftpa*, *Sftpb*, *Sftpc*, *Nkx2.1* and *β-actin* in AT1.1, AT2.1, MLE15 and adult lungs. The amplified products were then visualized by gel electrophoresis. (**D**) QRT-PCR was used to detect AT1 and AT2-specific genes in AT1.1 cells, MLE15 cells, and postnatal day 7 (PND7) mouse lungs. Data for genes expressed by AT2 cells is graphed as fold change relative to MLE15 cells ± standard deviation. $n = 3$ per condition. (*Sftpa1*: MLE15 vs PND7: $P = 0.0005$; *Sftpb*: MLE15 vs PND7: $P = 0.0005$; *Sftpc*: MLE15 vs PND7: $P < 0.0001$; *Sftpd*: MLE15 vs PND7: $P = 0.0011$). Data for genes expressed by AT1 cells is graphed as fold change relative to AT1.1 cells ± standard deviation. (*T1a*: AT1.1 vs PND7: $P = 0.3259$; *Aqp5*: AT1.1 vs PND7: $P = 0.0913$; *Hopx*: AT1.1 vs MLE15: $P = 0.0022$; AT1.1 vs PND7: $P = 0.1148$; MLE15 vs PND7: $P = 0.0245$; *Ifgbp2*: AT1.1 vs MLE15: $P = 0.0004$; AT1.1 vs PND7: $P = 0.2554$; MLE15 vs PND7: $P = 0.0016$; *Gramd2*: At1.1 vs PND7: $P = 0.1933$). Data reflect biological replicates analyzed by one-way ANOVA using Tukey-Kramer HSD (**D**).

© The Author(s)
